# Understanding attitudes toward adolescent vaccination and the decision-making dynamic among adolescents, parents and providers

**DOI:** 10.1186/1471-2458-12-509

**Published:** 2012-07-07

**Authors:** Charitha Gowda, Sarah E Schaffer, Kevin J Dombkowski, Amanda F Dempsey

**Affiliations:** 1 Child Health Evaluation and Research Unit, Department of Pediatrics, University of Michigan, 300 North Ingalls Building, Ann Arbor, MI, 48109-5456, USA; 2 Children’s Outcomes Research Program, University of Colorado Denver, 13199 E. Montview Blvd, Suite 300, Aurora, CO, 80238, USA

## Abstract

**Background:**

With several new vaccine recommendations specifically targeting adolescents, improving adolescent vaccination rates has become a major health priority. Vaccination attitudes are an important, modifiable target for new interventions. Prior research has examined primarily the attitudes and beliefs of adolescents, parents or healthcare providers separately without exploring the decision-making dynamic among these stakeholders. We sought to identify potentially modifiable barriers in the vaccine decision process among adolescents, parents and healthcare providers that could be addressed through interventions implemented within the adolescent’s medical home.

**Methods:**

We conducted a qualitative study of adolescents, their parents and healthcare providers, recruited from four primary care practices in Michigan. For each practice, three separate focus group discussions (adolescents, parents and healthcare providers, for a total of 12 focus groups) were conducted to explore vaccination attitudes, possible interventions to improve vaccine uptake and access to and use of technology for vaccination interventions. Themes that emerged from the focus group discussions were categorized using an inductive, iterative process, and analysis focused on highlighting similarities and differences among the three perspectives.

**Results:**

Participants included 32 adolescents, 33 parents and 28 providers. The majority of parents and adolescents were female. Lack of knowledge about recommended adolescent vaccinations was universally recognized among the three groups and was perceived to be the underlying driver of low immunization rates. Notably, each group did not appear to fully appreciate the challenges faced by the other stakeholders with respect to adolescent vaccination. Adolescents were seen as having a greater role in the vaccine decision-making dynamic than previously suggested. Provider-based interventions such as educational tools and reminder-recall notices were identified as important components of any immunization program. Overall, there was high receptivity among all stakeholders toward integrating technology such as email and Internet into new vaccination interventions.

**Conclusions:**

We identified potentially modifiable attitudinal barriers to adolescent vaccination among the three key stakeholders. However, there were notable differences in attitudes and preferences across the three perspectives, indicating that for an intervention to be successful it will require a dynamic partnership with the target audiences.

## Background

Over the last decade, several new vaccines, including the meningococcal conjugate (MCV4), tetanus-diphtheria-acellular pertussis (Tdap), human papillomavirus (HPV), and most recently seasonal influenza (Flu) vaccines, have been recommended for adolescents, making vaccination a major component of adolescent primary health care [1-4]. Despite their importance, in the United States immunization rates for adolescent vaccines lag behind those of childhood vaccines [5-7]. For example, coverage levels for childhood immunization against poliovirus, hepatitis B and varicella were above 90% in 2010 [6]. In contrast, although MCV4 has been recommended for adolescents since 2005 [1] and Tdap since 2006 [2], only 69% and 62% of all adolescents, respectively, had received the vaccines as of 2010 [5]. Far fewer adolescents, 35% [7] and 49%[5], respectively, have been vaccinated against Flu or begun the HPV series (among girls) as of 2010.

Barriers to achieving high vaccination rates are multifactorial. Inductive analyses have demonstrated that some of the most common barriers to vaccination include lack of education about vaccines and vaccine-preventable diseases, infrastructural issues, financial concerns, and the attitudes of adolescents, parents, and providers toward vaccination [8-11]. This complex interplay between vaccination coverage and individual, population and health system determinants is depicted in a conceptual model developed by Briss *et al.* (2000). This model not only illustrates commonly identified factors influencing vaccination coverage but also categories for health interventions that are posited to have beneficial public health outcomes if implemented [12].

Focusing on the individual-level determinants, several qualitative studies previously conducted have identified important parental or provider attitudes that may be modifiable to increase community demand for and access to vaccinations [8,10,13-15]. However, previous studies have not focused much attention on the adolescent perspective or the dynamic relationships among all three relevant stakeholders (adolescent, parent and provider) in influencing vaccination decisions.

With this in mind, we conducted a qualitative study of adolescents, their parents and the adolescents’ healthcare providers to identify similarities and differences in vaccination attitudes and practices among these groups and to explore the role of each stakeholder in the vaccine decision process. We then queried these parties for *their* ideas on possible interventions to improve vaccine uptake that addressed identified barriers in the vaccine decision process. Special consideration was given to exploring receptiveness among these stakeholders for using new technology (e.g. email and text messaging) given the growing integration of these technologies into everyday life [16-20].

## Methods

We conducted a qualitative study of adolescents, their parents and healthcare providers recruited from four primary care practices in Michigan. Focus group discussions were conducted to explore vaccination knowledge, attitudes, and practices and identify possible future interventions aimed at improving vaccine uptake among adolescents. All study activities were approved by the University of Michigan Medical School’s Institutional Review Board (IRB study protocol # HUM00043508).

### Healthcare provider recruitment

We recruited a convenience sample of four practices among the 10 practices with the largest volumes of adolescent patients from two counties in Michigan. The four practices varied in terms of geographic location within the state, immunization practices, uptake of vaccines among adolescents, and demographic characteristics of their adolescent patient population. Two practices were recruited from a county served primarily by suburban practices with relatively high immunization rates. The other two practices served mostly lower-income patients, were from a large metropolitan area and had lower immunization rates. For each of the practices recruited, we conducted three separate focus groups – that of adolescent patients in the practice, their parents, and their healthcare providers.

### Adolescent and parent recruitment

To recruit adolescent and parent participants, a random sample of 100 adolescents aged 11 – 18 years were identified from each practice using a computer randomization algorithm applied to electronic patient records. The parents of these adolescents received a letter outlining the study goals and time needed for participation. Interested parents contacted the study team member to determine a date and time for the focus group meetings. Each focus group was limited to ~12 participants (range 6 – 13 participants) so as to facilitate all members participating in the discussion. All parents who were able to read and converse in English and expressed interest in participating were selected to attend the focus groups until a group size of ~12 participants had been achieved. For inclusion in the adolescent focus groups, adolescents had to be able to read and converse in English and have obtained parental consent for their participation in addition to their written assent.

### Adolescent and parent focus groups

Study team members facilitated separate focus group discussions for adolescents and parents within each practice, for a total of 8 focus groups (4 parent, 4 adolescent). A focus group interview guide developed by the investigators was used to prompt discussion on specific topics including: barriers to adolescent vaccination, possible interventions, options for reminder-recall notices, the technical capacity of parents and adolescent patients, and priority areas for adolescent immunization. Adolescent and parent focus groups were conducted in separate spaces but at the same time and clinic location. Focus groups were allocated one hour for completion. Parent focus groups lasted approximately 45 minutes, and adolescent focus groups lasted approximately 20 minutes. Each participant received a $25 gift card (maximum $50/family) for their time.

### Provider focus groups

Using the previously developed interview guide to prompt discussion, a member of the study team facilitated 1-hour focus group discussions with providers from each participating practice. Any provider identified as being involved in immunization was invited to attend; thus, these groups included doctors, nurses, medical assistants, and other clinical staff. No incentives were given to the providers for their participation in the study.

### Data analysis

All focus group discussions were conducted in English and without gender disaggregation. One study team member moderated each focus group and the sessions were audio-taped with participant consent. These discussions were transcribed verbatim by an independent transcription service. Specific focus group participants were not identified in the transcripts. A thematic, inductive approach was used for data analysis. Transcripts were reviewed and coded by three members of the study team to group responses into thematic categories using an iterative process. Discrepancies in coding of themes were resolved by mutual agreement among the three reviewers. Analysis focused on highlighting the similarities and differences among the adolescents, parents, and providers from each practice.

## Results

Across the four practices, 32 adolescents, 33 parents and 28 healthcare providers participated in the focus groups (Table 1). The healthcare providers were roughly evenly distributed between physicians and supporting staff. The majority of participating adolescents and their parents were female.

**Table 1:** Composition of focus groups

Location*	**Adolescents**			**Parents**		**Healthcare Providers**			
	**Females**	**Males**	**Age Range**	**Mothers**	**Fathers**	**Physicians**	**Nurses**	**Clinic Coordinators**	**Medical Assistants**
Clinic #1	3	3	12 – 18 years	5	1	1	1	0	0
Clinic #2	6	3	11 – 16 years	4	3	5	4	2	0
Clinic #3	8	2	12 – 16 years	12	1	1	2	1	0
Clinic #4	5	2	11 – 18 years	7	0	4	2	1	4
Total # of participants	22	10	--	28	5	11	9	4	4

* Clinics #1 and #2 are two practices recruited from a county served primarily by suburban practices with relatively high immunization rates. The other two practices, Clinics #3 and #4, were from a large metropolitan area serving mostly lower-income patients with lower immunization rates.

### CURRENT BARRIERS TO ADOLESCENT VACCINATION

In focus group discussions, significant time was spent exploring what each group of stakeholders identified as the major barriers contributing to low adolescent vaccination levels. These barriers could be grouped broadly into two categories: 1) knowledge about adolescent vaccination, which included lack of awareness about recommendations for adolescents, impact of changing immunization schedules and concerns about vaccine safety; and 2) lack of routine preventive care among adolescents.

### Lack of awareness about recommended vaccines

Most parents and adolescents (from all four medical practices) were unaware that certain vaccines were recommended specifically for adolescents. At three of the four sites the majority of parents reported that they often did not have sufficient information about the vaccines to make vaccination decisions. Some parents expressed concerns about the information provided by their children’s doctors, citing that the information was biased toward promoting vaccine benefits while side effects were inadequately presented. Many of these parents reported seeking out other information sources including relatives or friends in the healthcare profession and Internet websites. The majority of parents at the fourth site believed they had enough knowledge about the vaccines to make informed decisions. However, these parents also shared that, for newer vaccines like the HPV vaccine, they had used online resources to acquire information. In contrast, most adolescents indicated that they did not need more vaccine information, stating instead that their parents informed them about upcoming vaccines as necessary. There was universal agreement among providers that parents and teens lacked understanding of adolescent vaccination recommendations. Table 2 highlights specific participant comments that exemplify these and other themes identified in our study.

**Table 2:** Current knowledge about and challenges to adolescent vaccination, as identified through focus group discussions

**Theme**		
**Sub-theme**	**Representative Quote from Provider**	**Representative Quote from Parent (P) or Adolescent (A)**
**Knowledge about adolescent vaccination**		
Lack of awareness about recommended adolescent vaccines	“I think it takes a lot of time in the office to go over each vaccine and to understand the importance of it, especially [with] new vaccines, you know, like the HPV vaccine … There’s a lack of understanding on their side.”	**A:** “I don’t know when we [should] get [vaccines] because most of the time my parents don’t either.”
		**A:** “Yeah, I never know when I have a vaccination coming. Until you get to [the doctor’s office] and they’re like, oh, you’re overdue.”
Impact of changing immunization schedules		**P:** “See the thing with me is they changed the vaccination rules … so I had to research all of this information in regard to shots. I didn’t know anything.”
		**P:** “Yeah, I feel like it’s easier to know what my dog needs than what my kids need.”
**Lack of routine preventive care**	“… a lot of education needs to be done in the community, letting them know that routine health is important … they’re not making it in for that routine visit, because the parents [think] the kid’s fine.”	**P:** “My daughter, up until this year, has not competed in sports and she’s healthy. So she hasn’t been to see the [doctor]. I called for, I don’t know, for some silly thing and I thought we could just call and get, I think it was a ‘script or something like that. And they said, ‘well, she probably should come in. It’s been ten years since she’s been in the office.’ And so all these, she had to have five shots. And it isn’t like I’m a bad mom…”
**Decision process about vaccination**		
Parents are primary decision makers	“I think a lot of parents let them get away with not wanting [the vaccines] … like the whiney 15-year-old who says she doesn’t want to do it, I think they are more likely to say, ‘ugh, she’s just being a teenager. Fine, we’ll just come back and do it.’ And then you sort of miss that opportunity.”	A: “I guess my parents mostly until, like, lately. They kinda tell me I need to get one and I’m OK with it.”
		P: “I made the decisions until my daughter was 18.”
Increasing role for adolescents in vaccination decisions	“Adolescents are more of a partner in their health decision-making than younger children, and they have the ability to say ‘no’, and parents will often respect that, rather than necessarily what the doctor is advising.	P: “I’ll let my daughter make the decision [to get the HPV vaccine] on her own.”

### Impact of changing immunization schedules

One of the barriers to adolescent vaccination readily identified by parents was changing immunization schedules. Parents at two sites specifically expressed frustration that vaccine recommendations and schedules are frequently changing, contributing to the difficulty of staying informed and leading to mistrust about whether vaccines were actually needed. For example, one parent did not believe that adolescents truly needed the Flu vaccine since it previously had not been recommended for them. Other parents felt that the recent implementation in Michigan of school mandates for Tdap and MCV4, but not HPV or Flu, vaccines suggested that only the mandated vaccines were important. While most adolescents did not express similar concerns, some did acknowledge that it was difficult for their parents to accurately know which vaccines were due and when. At one site, most of the adolescents reported that they and their parents relied primarily on their doctors to educate and remind them about vaccines.

Unlike parents, changing vaccine requirements was not mentioned by any of the providers as a barrier to adolescent vaccination, suggesting that providers may not recognize that these changes contribute to parental vaccination hesitancy. In addition, providers reported that school-based vaccine mandates appeared to improve coverage levels of all vaccines, even though parents indicated that they had differential attitudes based on whether vaccines were mandated or not.

### Concerns about vaccine safety

Most adolescents and parents expressed concerns over vaccine side effects. Adolescents tended to focus their concerns on more immediate side effects such as pain at the injection site, whereas parents were more focused on longer-term health consequences, as expressed specifically by parents from three sites. Parents at the fourth site stated that they did not have any specific concerns over the recommended vaccines but could understand how vaccine safety concerns might influence a parent to not vaccinate his or her child. Parental concerns included the use of thimerosal or aluminum in vaccine production and vaccine side effects such as seizures. Only a few parents at two sites mentioned that they were concerned about the link between vaccines and autism. Parents at all four sites had more reservations specifically about the effectiveness of the HPV and Flu vaccines. Regarding the HPV vaccine, parents wondered why the vaccine included only some and not all of the HPV strains and for how long the vaccine would provide protection. Personal experiences with the Flu vaccine had caused some parents to question its effectiveness and avoid having their children receive that vaccine. While providers universally recognized that concerns over safety and side effects from both adolescent and parent perspectives were significant barriers to adolescent vaccination, they reported generally not having adequate time to fully discuss these concerns during clinic visits.

### Lack of routine preventive care

Providers at all four sites reported that one of the largest barriers to adolescent vaccination is that adolescents are not seen routinely in the clinic, typically coming in only for urgent-care appointments. Parents agreed that their teens did not often visit the doctor; however, several parents indicated that the reason for the lack of visits was because they were actually unaware that annual preventive care visits and specific vaccines were recommended for adolescents. One parent even reported that, because her daughter was generally healthy and did not participate in sports requiring yearly physicals, she had not taken her child to see the doctor in 10 years. She was shocked to find out her daughter was behind on immunizations, leading to guilt about being a “bad mother.” According to most providers, when adolescents *do* come in for urgent visits, parents are hesitant to have their children vaccinated while currently ill. In addition, some providers reported that adolescents may come unaccompanied to their doctors’ visits and thus parental consent cannot be obtained for vaccination. Several adolescents indicated they would not want to get vaccinated unless a parent was present, and most parents shared this view.

### Decision-making dynamics about vaccinations

There was consistency between parents and adolescents when asked about the vaccine decision-making process – most agreed that parents were the primary decision-makers. One adolescent noted that she has gained more input in decision-making as she has gotten older, and several other adolescents at three sites stated that decisions are made jointly between their parents and them. However, the same participants also stated that their parents’ input receives more weight in the final vaccination decision. Some adolescents at two sites stated that they get all of the vaccines recommended by their doctor. At least two parents at two sites expressed different approaches with respect to the HPV vaccine, stating that they would leave that decision entirely up to their children.

These views contrasted with what most providers described as a tendency for parents to be easily swayed away from vaccination by their adolescents’ attitudes. Providers at most of the sites felt that teens were less likely to get vaccines in part because parents did not force vaccination decisions if a teen complained about anticipated pain or swelling from the vaccine. Several providers indicated that they wished parents would state more decisively that their teens needed to get vaccinated, as parents did with their younger children. At the same time, these providers acknowledged that there are differences in adolescents’ capacity to make decisions and perhaps teens should have more input in the decision process as they advance in age.

### Possible interventions to improve adolescent vaccination

**Figure 1:**
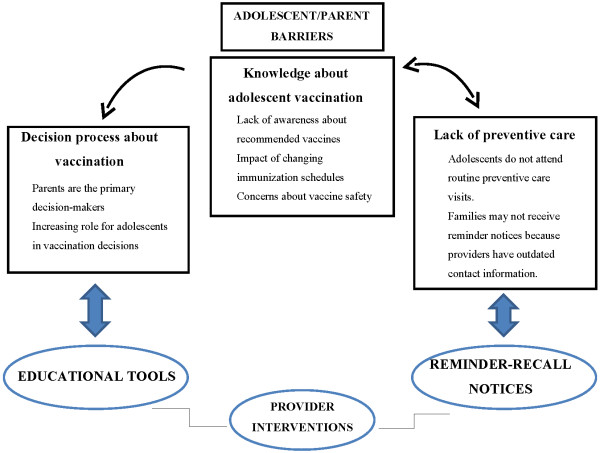
Conceptual model depicting proposed interventions to identified barriers in order to improve adolescent vaccination.

Parents and providers were asked to “brainstorm” about potential intervention options to improve adolescent vaccination. Two themes emerged that specifically addressed the main categories of barriers identified earlier: 1) improved educational tools to increase knowledge about adolescent vaccination and facilitate adolescent-parent discussion in the decision-making process; and 2) reminder-recall notices to improve adolescent participation in routine preventive care. In addition, the use of technology to facilitate both intervention strategies was explored. Figure 1 expands on the conceptual model developed by Briss *et al.* (2000)[12] to depict how identified individual-level barriers can be addressed with interventions proposed within the medical home that aim to increase community demand for and enhance access to vaccination. Table 3 presents participant comments that illustrate stakeholder interest in potential educational and reminder-recall intervention options.

**Table 3:** Strategies to improve adolescent vaccination rates including the use of new technologies, as identified through focus group discussions

**Theme**		
**Sub-theme**	**Representative Quote from Provider**	**Representative Quote from Parent (P) or Adolescent (A)**
**Educational tools**	“So if there was some way that’s in the media to increase the education before [the parents] came in. Because a lot of times they don’t know, and so once they come in, they’ll listen to you tell them about it, but then it’s, ‘Ok, we’ll think about it.’”	**P:** “… if you had all that information, you know, instead of walking into the office and them saying, ‘this is what’s due.’ You know, if you got that prior, I mean, obviously, that would be helpful.”
		**P:** “I was thinking, like, a webpage … giving us information about infants, you know, toddlers, young adults, or young adolescents. Giving us a chance to turn on, getting on, OK, well, my son’s 14-years-old. What is new out there for us?”
**Reminder-recall notices**	“Through the last couple of years, we’ve started printing our list of overdue teens and sending them [a] postcard … I think we’ve gotten a fairly good response from them … I think we started the year at 57% for our teen [immunization] rate, and we’re at like 75% over the course of the year.” “We’re still using a [database system] that … is not as smart as it could be … It could be embedded with more intelligence to pull out, you know, teenagers who are due for a tetanus shot or something, but the technology’s not there yet.”	**P:** “I get text messages from my kids but I, honestly, … would rather get a postcard.”
		**P:** “Ask preferences. You know, I prefer a postcard, but somebody … that’s 24 years old that has a new baby is probably gonna prefer a text message … I think you have to kinda look at different age groups and what they prefer.”
**Using technology**		
Varying levels of access to and comfort with using Internet	“We have a mixed population out here. Either there’s money to have [the Internet] or there’s not…”	**P:** “…it would be nice if there was a site where you can go and have all that information. ‘Cause I’m sure if you don’t have Internet at home, you can go to the library and access it somehow.”
	“Another suggestion for people that didn’t have access to the, to Internet, maybe we could have a computer [in the clinic] with information.”	
		**P:** “The people I talk to … a lot of them use Internet to access stuff regarding personal issues with their children
		and their families, and I think that would be wonderful.”
Using alternative communication modalities	“… I mean, they’re on their smart phones all the time, and Facebook, and texting … I think people would definitely be up for [using technology to communicate with the doctor].”	**P:** “Yeah, have all the modes of media because, you know, for most of us, this is, the phone is our lifeline.”
		**P:** “I mean, everybody’s got email now…”

### Educational interventions

Each practice currently employed different approaches to provide vaccine information to their patients and families. Most providers reported distributing educational material including vaccine-specific vaccine information statements (VIS) developed by the Centers for Disease Control and Prevention (CDC) or other general health brochures. One practice brought in a pharmaceutical representative to present information on the HPV vaccine to their patients. Another practice reported playing a television channel devoted to health issues (including vaccination) in the clinic waiting room, which parents had found informative.

Both parents and providers believed that it would be helpful to have additional vaccine-specific informational resources to offer to their patients. There was a particular desire in both groups to have the information provided *before* a scheduled visit so that there would be adequate time to review the materials. Many providers felt this could save time during appointments and meet the perceived need of many parents to have more information than providers may have time to convey. Parents at all four sites expressed interest in receiving emails with links to online vaccine-related resources prior to their child’s visit. Some parents were also open to browsing through vaccine-related information in the clinic waiting room, though other parents preferred to do that at home.

### Reminder-recall notices

Healthcare providers discussed a variety of strategies already employed by their practices to improve adolescent immunization rates. Three of the sites had taken advantage of the Michigan Care Improvement Registry (MCIR), Michigan’s statewide immunization registry, to access each patient’s vaccination status and provide that information to parents and patients at well child visits. At least two of the practices planned to expand their use of the MCIR to conduct assessments at all urgent care visits as well. Though the MCIR compiles records from >95% of primary care providers serving children throughout the state,[21] one practice reported that it did not consistently use the MCIR. This was despite its providers specifically commenting that their patients’ immunization records were located in multiple, disparate systems and the perceived lack of a central data repository made the tracking and assessment of vaccination status extremely difficult.

In addition to the use of the MCIR to determine patients’ vaccination statuses, most practices also conducted systematic reminder-recall notices – primarily by mailing letters or cards reminding patients about upcoming or overdue appointments although some practices also used reminder phone calls. Despite all of the practices’ efforts, the majority of parents interviewed felt that their providers did not adequately inform them when adolescents were due for vaccines.

Of note, practices did indicate that mail or phone reminder strategies were time-consuming and often unsuccessful as addresses or phone numbers were no longer valid at the time contact was attempted. These difficulties in reaching patients and their families due to incomplete or inaccurate demographic information likely contributed to the perception of inadequate provider communication expressed by parents who were unaware that providers faced such challenges.

### Using technology – email, texting and internet

There were mixed responses among the stakeholders when asked about Internet access and availability in their respective populations. While most healthcare providers thought that parents of adolescents knew how to use the Internet, they felt many parents would not have sufficient access or familiarity with this tool to use it regularly. In contrast, most parents reported having both Internet familiarity and regular accessibility, although this was not universal. Nonetheless, most parents, regardless of their Internet access or experience, were supportive of the idea of receiving e-mails from their children’s providers, either for reminder-recall purposes or with online links to medically-accurate, vaccine-related educational resources.

Providers at all four sites responded positively to the idea of using text messaging as a potential communication or reminder tool. However, one provider added the caveat that parents may have limited cell phone minutes or text messages permitted per month, which could hinder the reliability and effectiveness of this technology. While a few parents were receptive to receiving reminders about upcoming vaccine due-dates in the form of text messages, most parents were not supportive of the idea. Adolescents were adamantly opposed to receiving texts about vaccines themselves, indicating that texting was mainly for “friends” and that it would be “weird” for them to receive such a message. In addition, most adolescents believed that, although their parents currently may use text messaging for personal reasons, their parents would prefer phone or mail reminders about upcoming appointments over text messages.

## Discussion

We found in our qualitative study of adolescents, parents and healthcare providers that, while broad themes emerged across stakeholders with respect to vaccination barriers and possible interventions, there were differences in preferred implementation strategies among the groups that should be of fundamental consideration in designing future interventions. Importantly, no singular strategy emerged as a consistent response to barriers identified by all three groups. Instead, we found that intervention strategies will likely need to be tailored within provider settings to reflect the specific concerns and preferences of patients and their families.

### Knowledge about adolescent vaccination

We found that the lack of awareness about routine preventive care visits was a major source of frustration for healthcare providers as it limits the opportunities available for providers to educate patients and their parents. Consistent with earlier studies, our findings indicate that parents and adolescents are uninformed or inadequately informed about the importance of routine preventive care visits and immunizations during adolescence.[8,9,18,22,23] At the same time, most parents were aware that they are inadequately informed about adolescent health care issues and overwhelmingly felt they do not have access to medically-reliable resources to close the gaps. Changing vaccine schedules appears to contribute to parental frustration as these changes make it difficult for parents to stay informed. In addition, these ongoing modifications to vaccine requirements, without parents being adequately informed of the reasons for such changes, have contributed to greater uncertainty among parents about the true importance of vaccines – a consequence that providers may not have fully appreciated for its negative impact on parents’ vaccination attitudes. Taken together these findings suggest that if public health and medical providers want to improve parental acceptance of adolescent vaccines they need to specifically communicate to their patient population when and why vaccine schedules are being adjusted.

### Concern about newer vaccines

Our findings indicate that the HPV and Flu vaccines were distinguished from the other vaccines as being particularly problematic by parents in the study. This could be due to several reasons, including that these are the newest vaccines recommended for adolescents, there are no school-based mandates requiring them, and these vaccines require multiple shots (i.e. 3-doses for HPV, new Flu vaccine annually) and thus are more inconvenient to administer [24-26]. While providers in our study were universally cognizant that parents have concerns unique to the HPV and Flu vaccines, there is a paucity of interventions specific to these vaccines that have proven successful in allaying parental concerns. Educational efforts about adolescent vaccines have tended to focus on promoting all four adolescent vaccines generically to avoid stigmatizing HPV and Flu vaccines as particularly problematic, dangerous or unnecessary. However, based on our results, providers may wish to single out these vaccines in order to emphasize relevant information and correct misconceptions. Unfortunately, our study suggests that a significant barrier to doing this is the shared perception among both providers and parents that there is inadequate time to review vaccine-related information during clinic visits [27]. Thus, future research should examine alternative strategies to supplement patient-provider communication about vaccines. As an example, all of the stakeholders interviewed were highly receptive to the idea of accessing vaccine-related information via the Internet – particularly if provided prior to clinic visits. With adequate time, parents could review the information and formulate any remaining questions for the provider to address directly at the upcoming visit.

### The vaccination decision process

In our study, we found that both parents and adolescents independently agreed that parents ultimately decide whether or not an adolescent will be vaccinated. However, providers expressed frustration that some parents are too permissive in letting the adolescent control the vaccination decision; and, more focused discussion revealed that all parties accepted that adolescents have an increasing role in the decision process with advancing age. Thus, recognizing that parents may not be able or willing to insist upon vaccination of vaccine-hesitant adolescents, vaccination efforts may need to incorporate methods that incentivize adolescents directly. This may be particularly true for the HPV vaccine as we found that many parents elected to leave the decision entirely up to their child specifically for this vaccine, but not others.

Our focus group discussions did not capture why the HPV vaccine may be viewed differently from the other vaccines with respect to the decision-making process. One possible hypothesis is that since the HPV vaccine targets a sexually transmitted infection parents would prefer to let their children decide about that vaccination conjointly with decisions about sexual behavior practices they make as an adult. Further research is needed to elucidate the reasoning behind the differential approaches to vaccine decision-making for the HPV vaccine compared to the other recommended adolescent vaccines.

Only a few studies have previously examined the dynamics that occur *between* adolescents and parents regarding vaccine decisions, generally concluding that many (but not all) adolescents look towards parental values and beliefs when considering vaccination [28-31]. Moreover, to our knowledge only one study has simultaneously evaluated the decision-making dynamic that occurs among adolescents, parents *and* providers with respect to vaccination [32]. Focusing on the HPV vaccine, this study by Hughes *et al.* (2011) found that clinicians largely took their cues from parental attitudes, choosing not to urge vaccine-hesitant mothers to reconsider their decisions to refuse or delay HPV vaccination. The study also indicated that adolescents had a primarily passive role in vaccine decision-making, with their concerns limited only to immediate vaccine side effects.

Our results contrast those findings by Hughes *et al.* since we found that providers and parents reported adolescents’ concerns did influence whether adolescents were vaccinated or not. This difference could be due to variation between the two studies in participants’ sociodemographic characteristics. For example, our study had a greater number of older teens, fewer African-Americans and included fathers and adolescent boys whereas the study by Hughes *et al.* focused specifically on mothers and their daughters. The role that adolescents play in the vaccine decision-making process likely varies across races, cultural groups or by child’s age. Thus, for vaccination campaigns to be most effective, it may be necessary to first understand who will be involved in the vaccination decision process before deciding on where to focus educational efforts (i.e. on parents, adolescents, or both).

### Differences in technical capacity

Although our study identified that different technologies could be potentially integrated into future interventions, this opportunity appeared to be underappreciated by healthcare providers. Most providers were concerned about the number of parents without Internet access, yet we found that parents in general were both Internet-savvy and that some actually preferred email as their primary communication modality. In contrast, text messaging as a communication modality was not widely accepted by parents and teens – a preference not recognized by providers.

These preferences are important to consider as interventions to address key barriers are developed. For example, one potential barrier we identified was the difference in perspectives regarding whether providers or parents should be responsible for knowing an adolescent’s current vaccination status. Parents in our focus groups expressed reliance upon providers to pro-actively convey information, yet providers expected parents to realize that a vaccination-related appointment should be scheduled. One possible solution may be the development of an electronic mechanism to allow providers and parents to see the same vaccination history and follow-up recommendations for a child. In practice, this electronic portal would not only emulate the function historically served by paper immunization cards provided during early childhood but would also provide the recommended schedule of future vaccines for the adolescent. In doing so, information would be simultaneously shared with both the parent and provider and could serve to strengthen the patient-provider partnership while ensuring the most up-to-date and accurate information.

### Limitations

As with any qualitative study, the goal is to generate hypotheses that can be tested in the future rather than aim for generalizability of findings beyond the study population. To capture a wide range of opinions, we purposely sought out practices with a diversity of beliefs and approaches at both the adolescent/parent and provider levels. The brevity of the adolescent focus groups may have limited the quality of data gathered about adolescent vaccination attitudes as most discussions were completed within 20 minutes, even though one hour was allotted for the activity. Furthermore, the focus groups were not intentionally disaggregated by gender, although almost all of the focus groups were composed primarily of female participants. Having the ability to engage with the study population about proposed health interventions based on the challenges identified earlier on in the focus group resulted in a better understanding of the relative importance of these challenges as well as recognition of potential unintended consequences of such interventions. These findings clearly demonstrate that any health intervention would benefit from a dynamic partnership with the target audience in order to tailor to the audience’s specific needs as well as utilize available technologies most effectively for that population.

## Conclusions

Using a qualitative approach, this study provides insight into the vaccine decision-making dynamic among adolescents, their parents and healthcare providers. We found several similarities in vaccine attitudes that could be used as foundations for future interventions. However, there were notable differences of opinions among these groups that will need to be considered when developing future interventions to ensure their effectiveness. Furthermore, the integration of technologies such as e-mail and the Internet may offer new strategies to address the perceived lack of comprehensive medical information available to parents as well as enhance direct communication between patient/parent and providers. A next step will be to use these findings to develop interventions that support the specific needs identified by each of these stakeholder groups.

## Abbreviations

CDC, Centers for Disease Control and Prevention; HPV, Human papillomavirus; MCV4, Meningococcal conjugate vaccine; MCIR, Michigan Care Improvement Registry; Tdap, Tetanus-diphtheria-acellular pertussis; VIS, Vaccine information statements.

## Competing interests

Since June 2009 Amanda Dempsey has served as an advisory board member for Merck, providing advice on male HPV vaccination. This company had no role in the design or analysis of this study and is unaware of the study’s results. Dr. Dempsey does not receive research support from this company. The remaining authors have no competing interests to declare.

## Authors’ contributions

CG participated in data analysis and contributed to drafting the manuscript. SES conducted focus groups, participated in data analysis and critically reviewed the manuscript. KJD helped conceive of the study, participated in data analysis and critically reviewed the manuscript. AFD conceived of the study, conducted focus groups, participated in data analysis, and contributed to drafting the manuscript. All authors read and approved of the final manuscript.

## Pre-publication history

The pre-publication history for this paper can be accessed here:

http://www.biomedcentral.com/1471-2458/12/509/prepub
